# Laser-assisted socket seal surgery using bioactive glass for dental implant site development: a randomized clinical trial

**DOI:** 10.1038/s41598-025-28824-7

**Published:** 2025-12-09

**Authors:** Hala H. Hazzaa, Marwa A. M. El Shiekh, Mena F. AbdAllah, Nora Abdelgawad, Mohamed A. El-Mahdy, Hazem Shawky Shoshan, Gasser M. Elewa

**Affiliations:** 1 Faculty of Dental Medicine for Girls, Periodontology and Diagnosis, Al Azhar University, Cairo, Egypt; 2 Faculty of Dental Medicine for Girls, Al Azhar University, Cairo, Egypt; 3 Faculty of Dental Medicine for Girls, Oral Biology, Al-Azhar University, Cairo, Egypt; 4 Faculty of Dental Medicine for Girls, Oral Medicine, Periodontology and Diagnosis, Al Azhar University, Cairo, Egypt; 5 Faculty of Dentistry, Al Nahda University, Benisuef, Egypt; 6 Faculty of Dentistry, Oral and Maxillofacial Department, Cairo University, New Cairo, Egypt; 7 Faculty of Oral and Dental Medicine, Delta University for Science and Technology, Dakahlia, Egypt

**Keywords:** Socket seal surgery, Photobiomodulation, Bioactive glass, Biotechnology, Diseases, Health care, Materials science, Medical research

## Abstract

Preserving the alveolar ridge after extraction is crucial for implants; different biomaterials and techniques allow predictable outcomes. This study aimed to assess laser photo-biomodulation (PBM) as a complement for socket seal surgery (SSS) when extraction sockets are grafted with bioactive glass and sealed by platelet-rich fibrin (PRF) membrane in humans. 45 sites were randomly and equally distributed into 3 groups: Control: PRF membrane for socket seal, with no more interference; it is a unified step in all groups, Test I: bioactive glass, and Test II: received the same as Test I plus PBM-irradiation. Cone-beam computed tomography (CBCT) was used to assess the dimensional changes in height and width measurements of the alveolar ridge at 0 and 9 months postoperatively. Bone samples were obtained from osteotomy sites at the time of implant placement, and then sent for histological staining using hematoxylin and eosin and Masson trichrome stain for assessment of newly formed bone and histomorphometric analysis in terms of percentage (%) of new bone surface area and % of unmineralized bone, respectively. The Control group revealed a significant reduction in alveolar ridge dimensions over time compared to the test groups (*P-value* < 0.001). Test II has shown less bone reduction over time, with a *P-value* < 0.001. Histological results revealed that Test II showed large areas of connected, highly mature bone trabeculae without any collagen fibers or immature matrix in between. Compared to the other two groups, Test II provided superior bone quality. PBM can be considered as a promising adjunctive therapy for socket seal approaches when combined with bioactive glass, as evidenced by the better clinical improvement and the remarkable histological findings for bone repair compared with the non-lased group.

*Trial registration:* The current randomized clinical trial was registered at ClinicalTrials.gov (Registration number: NCT06764732) and it was released on 8/1 /2025**.**

## Introduction

The management of post-extraction socket constitutes a challenging part in clinical dentistry, especially if dental implants are planned. Maintenance of stable ridge dimensions is now considered important to simplify post-extraction protocols and enhance their outcomes^1^. Socket Seal Surgery (SSS) is an alternative for the management of the extraction socket that requires a primary closure of the wound to conserve the form and volume of the soft tissues and the jawbone. It can complement the guided bone regeneration (GBR), protect the graft from being infected, and preserve the ridge from collapsing^2^. Currently, platelet-rich fibrin (PRF) can be used to seal post-extraction sockets with the advantages of lower surgical time, less postoperative pain, and less scarring^3^.

Focusing on oral application, bone substitute materials have become popular owing to their low postoperative morbidity and the unlimited amount available. In this area, bioactive glass was reported as a promising scaffolding material, being a time-tested regenerative biomaterial that has osteoconductive properties with adequate capability of bone formation^4,5^.

Bioactive glass consists of calcium oxide, sodium oxide, phosphorus pentoxide, silicon dioxide, and silica. These materials can bind chemically with bone, forming a biologically active hydrated calcium phosphate layer at the surface of the bioactive glass. It has the advantages of enhanced biocompatibility, resorption at the right time, ion leaching properties, and the unique ability to support the migration and proliferation of osteogenic cells^5^.

In spite of that, the body’s natural regenerative capacity can be compromised due to certain variables such as the severity of the injury and its location. In addition to the systemic limitations, necessitating therapeutic interventions to promote effective bone healing.

In view of the search for an efficient structural and functional remodelling pattern of lost tissues, photobiomodulation (PBM) is a non-invasive technique to stimulate cellular processes promoting tissue repair and regeneration^6^. Photobiomodulation utilizes low-intensity lasers to elicit photo-physical and photo-chemical reactions, as well as generate biostimulatory effects^7^. In the early stage of wound healing, PBM encourages the production of pro-angiogenic growth factors, facilitates angiogenesis, and enhances the migration and proliferation of endothelial cells^8^. Moreover, PBM exerts an initiating action in accelerating wound healing and promoting bone regeneration through a unique action in stimulating the proliferation of osteoblasts and fibroblasts^9^.

PBM has been recommended as a good tool to promote beneficial effects on the repair of critical-sized bone defects^10^. Nevertheless, PBM has certain limitations which should be properly justified, including the lack of PBM to the physical support required for the biological stimulation of the tissue regeneration sequences^11^. Consequently, biomaterials are considered crucial in promoting an ideal bone defect repair scenario.

The integration of PBM with biomaterials has gained increasing awareness in tissue engineering and GBR to maximize clinical benefits^12^. In this item, their combination was urged to enhance regenerative outcomes, as evidenced by the pre-clinical conclusions of Pinheiro et al.^13,14^, who demonstrated improved bone mineral density and graft integration. Taking the relevant criteria of bioactive glass and PBM into consideration, the authors hypothesized that their combined application would have an additive effect in the enhancement of bone fill.

Therefore, this study aimed to evaluate a novel ridge preservation approach (Laser-assisted-SSS), which focuses on preserving socket dimensions following extraction, using PBM as an adjunct to bioactive glass in sockets sealed with PRF, compared to using bioactive glass alone. The primary outcome was alveolar ridge dimensional changes assessed through cone-beam computed tomography (CBCT) at baseline and 9 months.

## Patients and methods

### Study design and population

This study was a randomized controlled clinical trial. Participants were taken at the time from November 2022 to February 2024. A total of 62 patients who asked for dental implant insertion after tooth extractions in the posterior maxilla and mandible were recruited at Outpatient’s Clinic, Faculty of Oral and Dental Medicine, Delta University for Science and Technology (DU), Dakahlia, Egypt. Patients fulfilling all inclusion criteria were invited (N = 45) to participate in this study, and received information about the study protocol and signed the informed consent form. The study protocol was approved by the research protocols of the Ethics Committee for Research (DU: 024,100,558) and conducted in accordance with the Helsinki Declaration of 1975, as revised in 2013.”

Inclusion Criteria:

- The patients’ age group > 18 years old.
- Patients were systematically healthy patients^15^.
- 4 mm of residual alveolar bone, at least, should be present from the borders for future prosthetic rehabilitation improving as recommended by various researchers^16,17^.
- Presence of at least a periodontally affected and non-restorable tooth in the posterior region (Class II sockets)^18^.
- Reasons for extraction were caries, endodontic complications, root fracture, or trauma.
- At least 10% plaque control scoring was accepted, as recorded by O’Leary^19^.
- A moderate to thick gingival phenotype^20^.

Exclusion Criteria:

- Patients with systemic conditions that may impair bone healing (e.g., Uncontrolled diabetes, thyroid gland disorders, vitamin D deficiency)
- History of chemotherapy, radiotherapy, or bisphosphonate therapy.
- Smoking or parafunctional habits that interfere with healing and osseointegration.
- Active periodontal or periapical infections at the extraction site.
- Pregnancy or lactation.
- Patients are unwilling to comply with post-operative care and follow-up visits.

Sample size calculation.

To study the socket preservation using a graft of bioactive glass either alone or combined with Laser Photobiomodulation (PBM) irradiation in periodontitis patients, a priori analysis was performed to compute the required sample size-given α, power, and effect size.

According to Rosero et al.^21^ a total sample size of 45 cases was sufficient to detect the effect size of 0.40, a power (1-β) of 80% and at a significant level of 5% (p < 0.05), each group would be represented by 15 cases. The sample size was calculated according to G*Power software version 3.1.9.4. Where, *f*S is the effect size, α = 0.05, β = 0.2, and Power = 1- β = 0.8.

### Patients’ randomization and grouping

Randomization was carried out by an independent researcher who was not involved in the study. A computer-generated random sequence was used to allocate the extraction sites equally into the study groups. Allocation concealment was ensured using sequentially numbered, opaque, sealed envelopes (SNOSE) containing the group assignments. The envelopes were opened after the patient’s eligibility had been confirmed. The independent researcher informed the surgeon of the assigned intervention for each site immediately before the procedure. Both the outcome assessor and the statistician were blinded to the allocation during the study period.

Each patient presented with a posterior tooth indicated for extraction. The extraction sites were randomly and equally allocated into 3 groups: 1) Control: 15 sockets were sealed with PRF membrane only. 2) Test I: 15 sockets were filled with bioactive glass graft and sealed with PRF membrane. 3) Test II: 15 sockets were filled with bioactive glass graft, sealed with PRF membrane, and subjected to PBM.

Platelet-rich fibrin membrane preparation: For each patient, 10 mL of venous blood was collected into sterile glass tubes (without anticoagulant) and immediately centrifuged using a table-top centrifuge (IntraSpin® system, Intra-Lock International, Boca Raton, FL, USA) at 2700 rpm (≈ 400 g) for 12 min at room temperature (22–24 °C). This process yielded three layers: acellular platelet-poor plasma at the top, the PRF clot in the middle, and the red blood cell fraction at the bottom. The PRF clot was carefully removed with sterile forceps, separated from the red blood cell layer with scissors, and gently compressed between two sterile gauze pieces for 1–2 min to form a membrane suitable for socket sealing. All steps were performed under strict aseptic conditions, and the time from blood draw to clot retrieval did not exceed 2 min to preserve fibrin integrity and cellular viability^22^.

Bioactive glass: Each gm contains calcium sodium phosphosilicate, forming a bioactive composition similar to standard 45S5 bioactive glass (SiO₂: 45%, Na₂O: 24.5%, CaO: 24.5%, P₂O₅: 6%). supplied as particulate resorbable form, (400-800um) particle size (Intentional Biotechnology CO, USA).

**Fig. 1:**
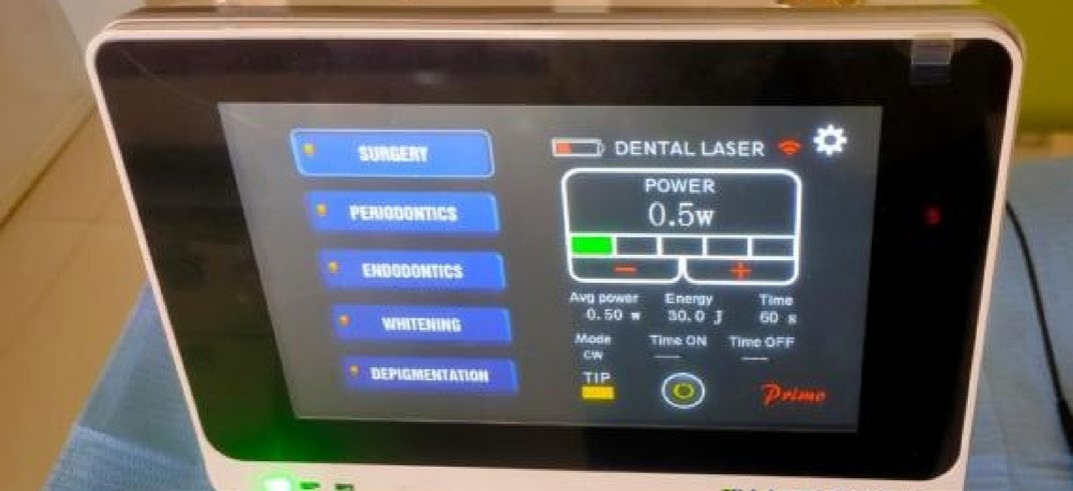
Laser device with applied parameters**.**

Laser irradiation protocol: In Test II, defects were treated with PBM (Primo diode laser by Medency, Vicenza, Italy). The used laser parameters were set as: wavelength, 980 nm; power, 0.50 W; energy, 30 J/point; power density, 0.69 W/cm^2^; fluence, 41.4 J/cm^2^ for 60 s through a spot size of 0.724 cm^2^ in continuous wave mode (CW) for each point of application in a non-contact mode (about 1 cm away from the target tissue). Three points of applications around the extraction socket (buccal, lingual, and occlusal) were irradiated immediately after tooth extraction (day 0) and at 48 h intervals for 15 days (i.e., on days 0,3,6,9, and 15) for a total of 6 sessions^23^ (Fig. 1).

### Surgical procedures

The same surgeon performed the surgical steps under local anesthesia using 2% mepivacaine HCL, combined with 1:1000 levonordefrin (Alexandria Company for Pharmaceutica) and sterile conditions. A minimally invasive flapless tooth extraction was done. The gingival fibers were cut with a scalpel, then a periotome (Hu-Friedy Mfg. Co., LLC, Chicago, IL, USA) was used for tooth luxation through periodontal ligament fibers cutting.

Next, atraumatic tooth extraction was performed for tooth removal, thus avoiding the usage of any elevators or forceps. Sites with bone loss in the buccal or palatal /lingual plate of < 50% of the initial bone height were included. The extraction sockets were curetted to remove any remaining granulation tissue, infected debris, or epithelial remnants that could interfere with bone healing and graft integration, irrigated with sterile saline.

Experimental sockets (Test I, Test II) were packed with the bioactive glass graft applied in particulate form, and the quantity was determined based on the socket size to ensure complete filling without over-packing. Given the anatomical variations between single-rooted and multi-rooted sockets, the total amount varied accordingly.

**Fig. 2:**
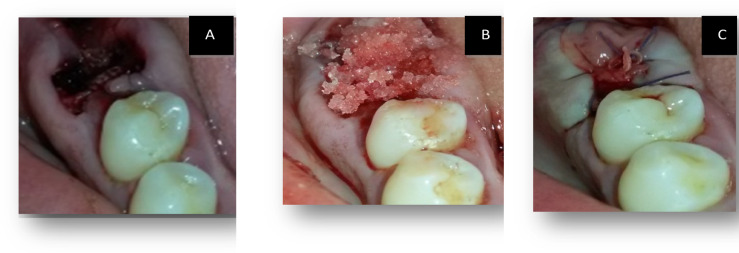
Clinical steps of socket management. (**a**) Extraction socket. (**b**) Application of bone graft after debridement. (**c**) Sealing of the socket entrance with a PRF membrane secured with sutures.

**Fig. 3:**
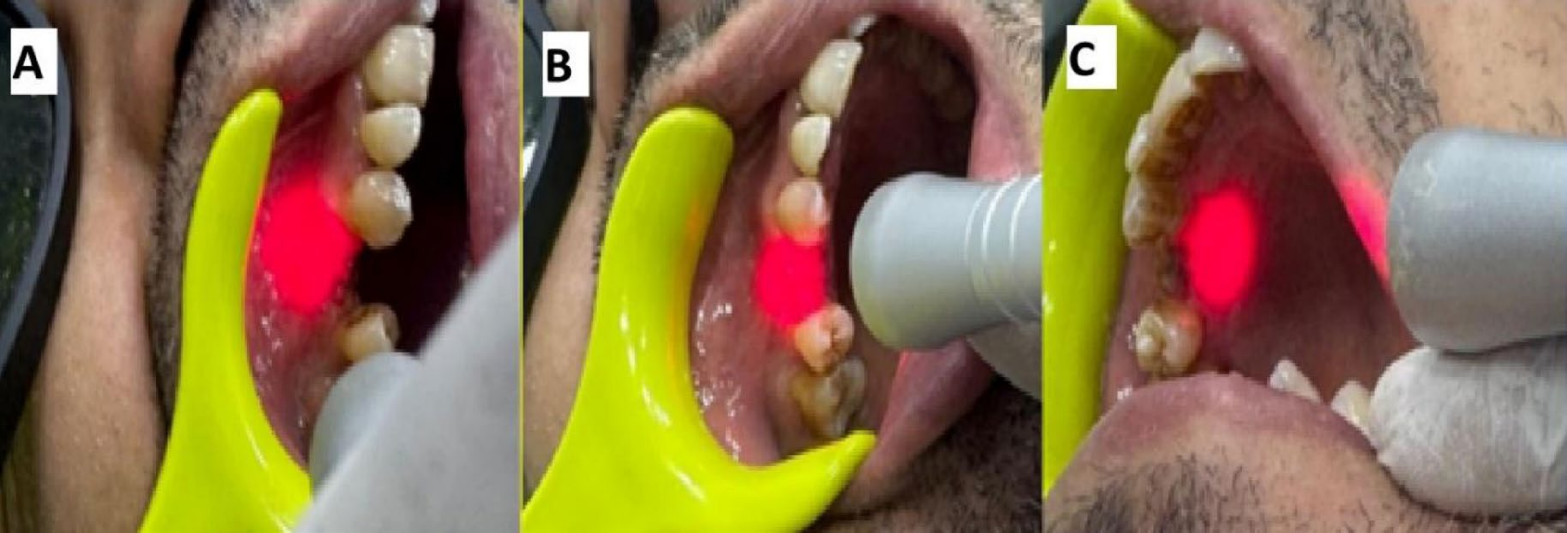
**.** Laser application at the extraction socket. (**a**) Buccal. (**b**) Occlusal. (**c**) Palatal.

All sockets (Control, Test I, and Test II) were sealed with PRF membrane and sutured using a crisscross technique with 5–0 polypropylene sutures (Eldawlia Tibbi Atik Medical, Istanbul, Turkey) (Fig. 2). PBM was immediately started according to the mentioned protocol in Test II. (Fig. 3). After socket preservation, patients were instructed to avoid trauma to the surgical site, not to disturb the sutures, and to refrain from consuming hot foods during the first 24 h. Antibiotic coverage included amoxicillin 500 mg capsules taken three times daily for one week. Ibuprofen 400 mg tablets were recommended twice daily for three days when needed to manage significant postoperative pain. Patients were advised to avoid brushing the surgical area for two weeks and to use a 0.12% chlorhexidine mouth rinse twice daily during this period to prevent infection. Gentle brushing at the surgical site was permitted after two weeks. Healing progress was monitored through weekly follow-up visits, and sutures were removed 14 days postoperatively.

All implants were placed at 9 months post-extraction, regardless of whether the socket was in the maxilla or mandible. This standardized healing period was chosen to allow adequate bone remodeling and ensure consistent evaluation of alveolar ridge preservation across all groups.

### Evaluation of the treatment outcome

1. Radiographic assessment:

The CBCT scan was taken using (Planmeca ProMax 3D classic, Helsinki, Finland), and Scans were exported in Digital Imaging and Communications in Medicine (DICOM) format to the (Planmeca Romexis dental software). The chins and heads of the patients were properly stabilized for 40 seconds to be scanned by CBCT (scan dimensions of 8.0x8.0 cm) with 0.2mm voxel size,90 KV, and 8 mA. The scans were done at baseline (before extraction) and repeated 9 months post-extraction.

Prior to extraction, each socket site was evaluated radiographically using CBCT images to determine the defect morphology and the initial buccal bone thickness. The defect morphology was classified according to the vertical position of the bone defect relative to the cementoenamel junction into coronal, middle, or apical types. This classification was adapted from Kim et al. (2021)^18^ to characterize the extent of hard tissue loss.

The initial buccal bone thickness was measured on the CBCT cross-sectional image as the horizontal distance between the outer buccal bone surface and the root surface at 1, 3, and 5 mm apical to the alveolar crest, and the mean values was recorded for each socket^24^.

**Fig. 4:**
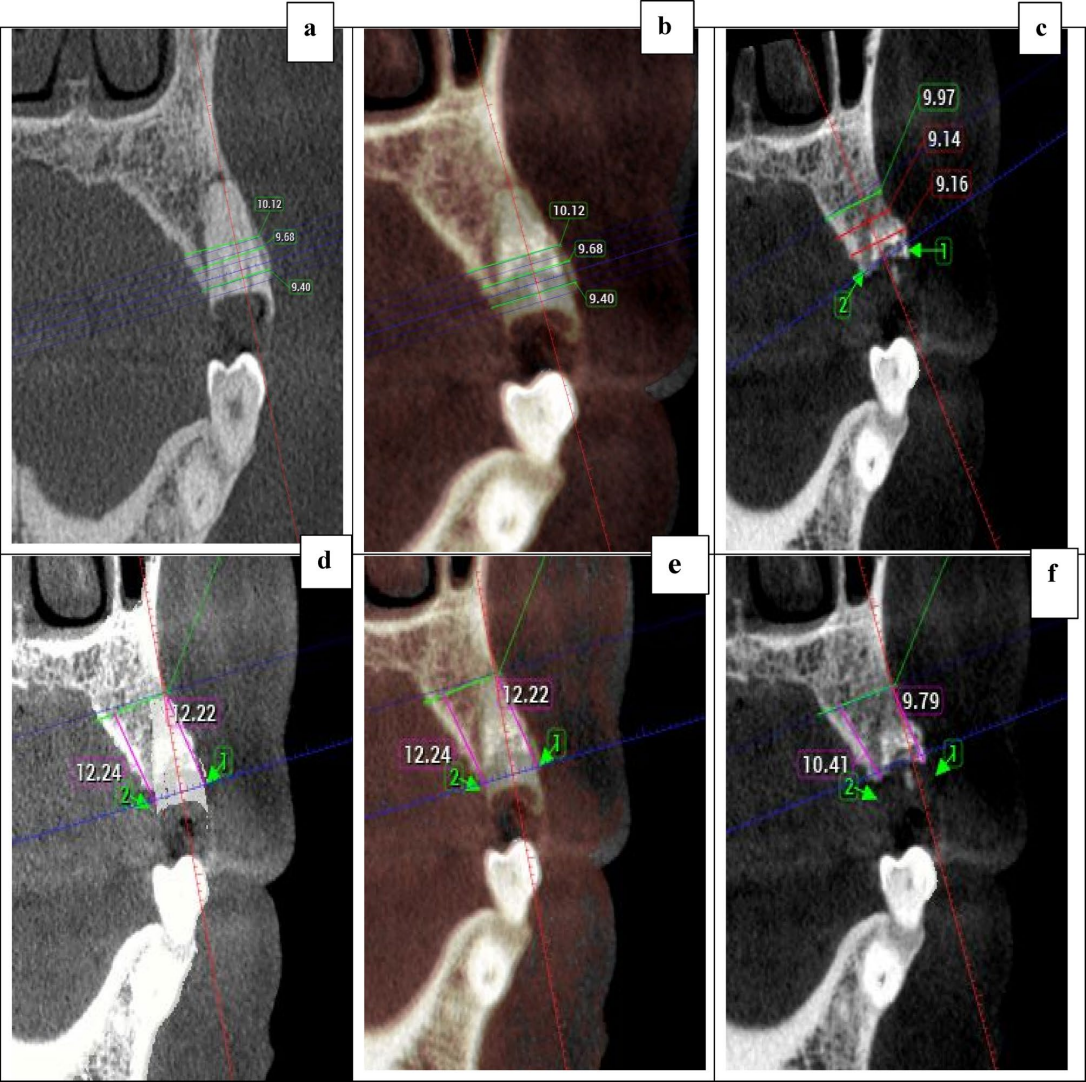
Cone-beam computed tomography (CBCT) evaluation., (**a**) initial CBCT scan with ridge width measurements at 1, 3 and 5 mm below the most coronal aspect of the crest, (**b**) fusion superimposition of the initial and 9 months post-operative scans, (**c**) 9 months postoperative scan, (**d**) initial CBCT scan showing a tangential line drawn at the most apical point of the socket depth parallel to the horizontal plane, points marked at the most crestal parts of the buccal and palatal ridges and line dropped to measure buccal and palatal ridge heights, (**e**) fusion superimposition of the initial and 9 months post-operative scans, (**f**) 9 months postoperative scan.

Preoperative images were first aligned with postoperative images using manual registration, followed by automatic registration based on anatomical landmarks of the jaw. Baseline measurements were obtained from the primary (preoperative) image. Thereafter, the measurements were cleared, and the primary image was deactivated, leaving only the secondary (postoperative) image. Measurements were then repeated on the secondary image using identical plane orientation, direction, and slice position as those of the primary image to ensure consistency and reproducibility. From the outer surface of the buccal and lingual/palatal ridges of the socket, the bucco palatal/lingual width was measured at three different levels (below the bone crest by 1, 3, and 5 mm). For alveolar ridge height measurement, a parallel tangential line was drawn to the horizontal line at the deepest point of the socket apex. From the highest crestal parts of buccal and lingual/palatal ridges, another line was dropped perpendicular to the initial line to determine their heights (Fig. 4).

The amount of alveolar ridge dimensions was calculated by deducting the final values from the baseline values and recorded in millimeters^25^.

2. Histological assessment:

At 9 months (the time of dental implant placement), bone biopsies (2 mm x 6 mm) were harvested from the osteotomy sites by a 3 mm surgical trephine (GDC, Punjab, India). Specimens were fixed in 10% buffered formalin for a week and then decalcified in formic acid–sodium nitrate solution. Paraffin tissue blocks prepared from the routinely processed specimens were cut into 5 to 7 mm slices and deparaffinized. After staining with hematoxylin and eosin (H&E), the sections were examined using a light microscope (Olympus BX60; Olympus Optical Co, Tokyo, Japan) attached to a color video camera connected to a personal computer. The images were captured at 40X and 100X magnification for examining bone quality. Other sections were stained using Masson trichrome special stain to differentiate areas of immature bone with newly formed collagen from mature bone areas^26^.

3. Histomorphometric analysis:

The histological sections were examined by L/M at 40X magnification to detect the new bone area % in H&E specimens by tracing the area of newly formed bone in relation to the total field. The un-mineralized bone area percentage (%) in Maison trichrome specimens at 40X magnification was also calculated, where green isolated areas were representative of the un-mineralized bone area % compared to the whole tested field. An image analyzer computer system using Leica Qwin 500 Software, Germany. It is composed of L/M, supplied with a digital camera, together with a computer, which has a high-speed digital image processing for measurements. The image analyzer was automatically calibrated to convert the pixels (measurement units) produced by the image analyzer program into actual micrometer units^27^. This process was carried out for 5 serial sections of each specimen, and a mean value was calculated.

Radiographic and histological evaluations were conducted by two independent examiners, both highly experienced specialists and not involved in the study. To ensure measurement reliability, inter-rater agreement was evaluated using the intraclass correlation coefficient (ICC), with all values exceeding 0.90, indicating excellent consistency between observers.

### Statistical analysis

One-way Analysis of Variance (ANOVA) followed by Tukey Post Hoc test was used for inter-group comparison between the three groups and intra-group analysis between the three levels (1, 3, and 5 mm). Paired T-test was used for inter-group comparison between the two sides of the study (buccal and palatal). *P*-value ≤ 0.05 was considered statistically significant (95% significance level), *P*-value ≤ 0.001 was considered highly statistically significant (99% significance level). The Shapiro–Wilk test was used to test the normality of the data. Statistical evaluation was performed using the SPSS statistical package (version 25, IBM Co., USA).

## Results

### Patients’ criteria

**Fig. 5:**
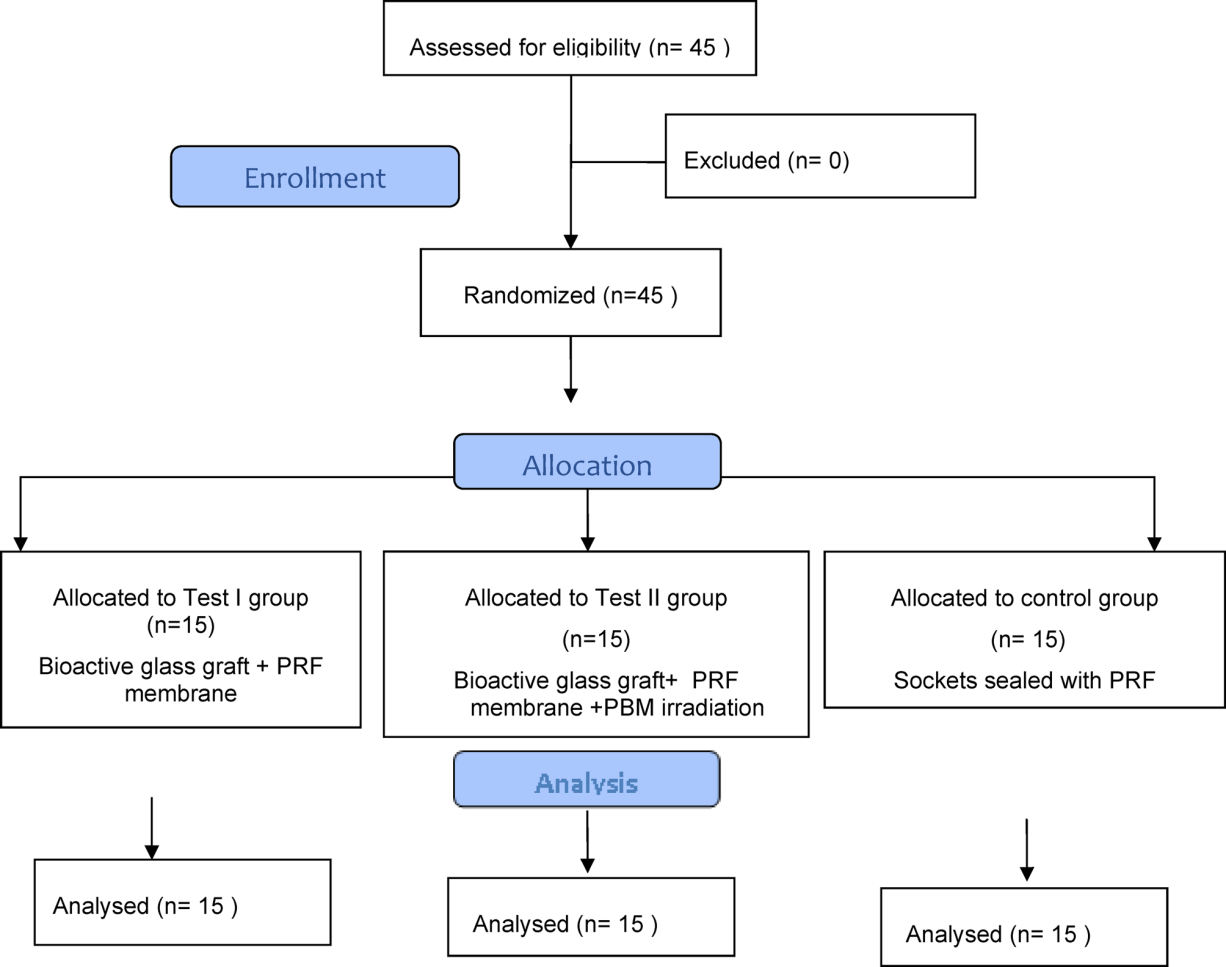
CONSORT flow chart of patient enrollment, allocation, follow-up, and analysis.

The patient distribution through each stage of the randomized clinical trial according to the CONSORT statement flow chart is reported in Fig. 5 where “n” represents the number of patients. In total, 45 sites (1 site/patient) were included in this study. 22 patients received extractions in the maxilla and 23 received extractions on the lower jaw. All extractions were located in the posterior region. In every patient, a proper examination was done for each site at the time of extraction to make sure of the socket type. All patients followed the study protocol and completed the follow-up period with no complaints or failure signs. Our patients reported negligible postoperative pain and discomfort, well-tolerated procedures, and no edema, with favorable healing outcomes.

### Demographic data

No statistically significant differences were observed among the study groups regarding age (p = 0.673), gender distribution (p = 0.703), surgical site (p = 0.544), defect morphology (p = 0.456), or initial buccal bone thickness (p = 0.123) (Table 1). All included extraction sockets exhibited a three-walled configuration with partial bone loss extended (coronal or middle), as verified radiographically using CBCT images before extraction.

**Table 1:** The mean and standard deviation (SD) of age and frequency & percentage of gender, site, defect morphology and initial buccal bone thickness for tested groups.

Variables		Demographic data						
		Control		Group I		Group II		*p*-value
		Mean/n	SD/%	Mean/n	SD/%	Mean/n	SD/%	
Age (Years)		41.20	4.49	42.40	4.45	40.87	5.76	0.673 ns
Gender (n, %)	Females	8	53.3%	6	40%	6	40%	0.703 ns
	Males	7	46.7%	9	60%	9	60%	
Site (n, %)	Maxilla	7	46.7%	9	60%	**6**	40%	0.544 ns
	Mandible	8	53.3%	6	40%	**9**	60%	
Defect morphology (n, %)	Coronal	11	73.3%	8	53.3%	9	60.0%	0.456 ns
	Middle	4	26.7%	7	46.7%	6	40.0%	
Initial buccal bone thickness (mm)		0.99	0.41	1.25	0.66	1.22	0.73	0.123 ns

Mean and SD of age were compared between three different groups using one way ANOVA test, while frequency and percentage of gender and site were compared between three different groups using Chi square test. ns; non-significant (p > 0.05).

### Study parameters

#### I. Radiographic assessment

- Assessment of the reduction in the alveolar ridge height

**Fig. 6:**
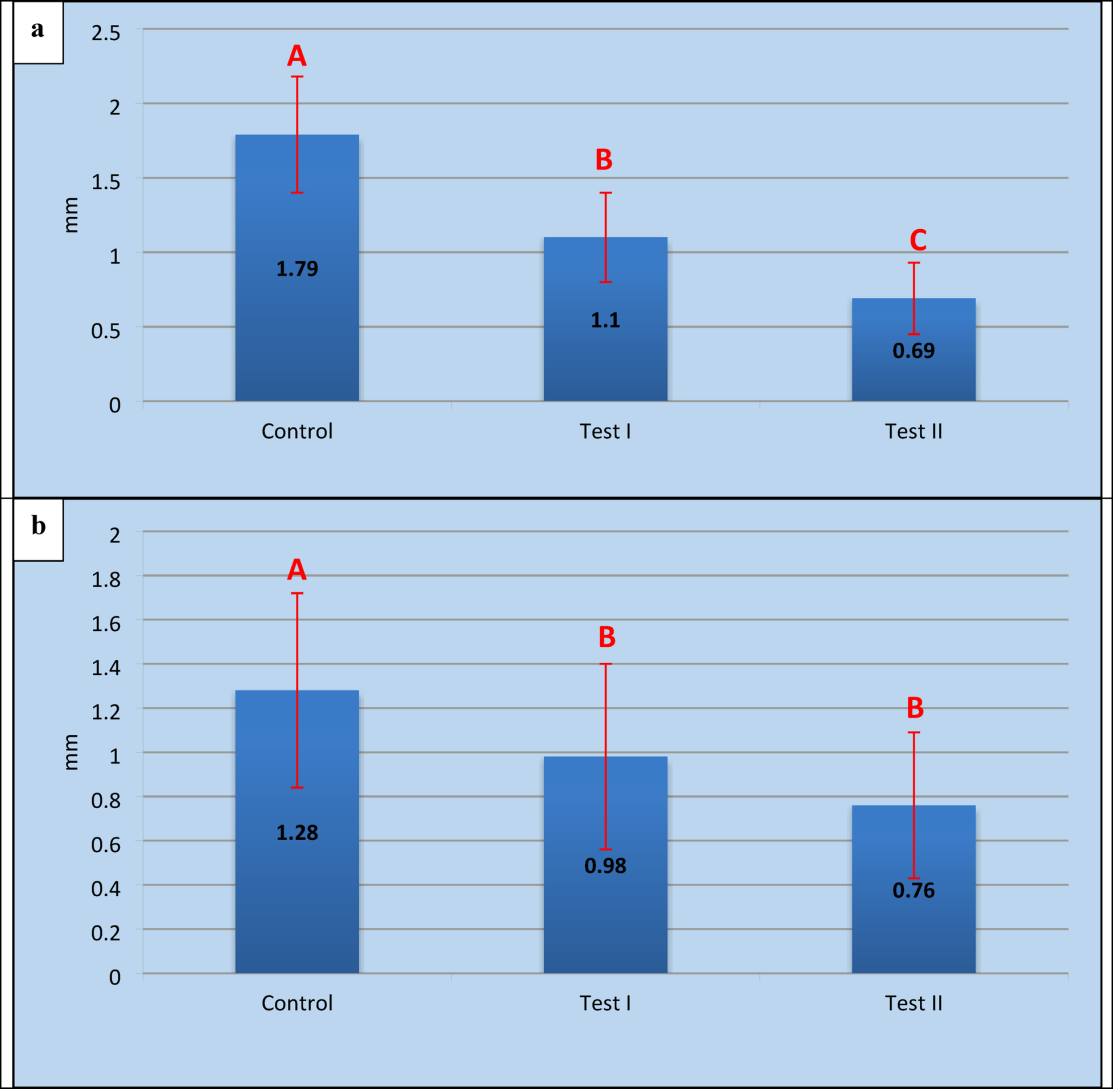
Alveolar ridge dimensional changes. (**a**) Mean reductions in ridge height for the three study groups. (**b**) Mean reductions in ridge width at 1, 3, and 5 mm levels for the three study groups.

The control group revealed the highest significant mean reduction in alveolar ridge height (*P-value* = 0.001) on the buccal side 2.07 ± 0.34 mm, decreasing to 1.52 ± 0.19 mm on the palatal/lingual side, with an average of 1.79 ± 0.39 mm between the two sides. In Test I, the mean reduction was 1.32 ± 0.2 mm on the buccal side, decreasing to 0.88 ± 0.21 mm at the lingual /palatal side, resulting in an average of 1.1 ± 0.30 mm (*P-value* = 0.021). Test II, showed the least reduction with an average 0.69 ± 0.24 mm (*P-value* = 0.422). The mean reduction was 0.72 ± 0.21 mm buccally, decreasing to 0.67 ± 0.29 mm lingually/palatally. For all three groups, the buccal side showed more resorption than the palatal /lingual side in all groups. Test II showed the statistically significant least reduction regarding the inter-group analysis (*P-value* ≤ 0.001) (Table 2, Fig. 6a).

**Table 2:** Mean ± SD, intra, and inter group comparison of reductions in alveolar ridge height measurements (mm) in buccal and palatal / lingual sides for the three studied groups.

	Buccal	Palatal / Lingual	*P*-Value*	Average
**Control**	2.07 ± 0.34^**A**^	1.52 ± 0.19^**A**^	0.001^**HS**^	1.79 ± 0.39^**A**^
**Test I**	1.32 ± 0.2^**B**^	0.88 ± 0.21^**B**^	0.021^**S**^	1.1 ± 0.30^**B**^
**Test II**	0.72 ± 0.21^**C**^	0.67 ± 0.29^**B**^	0.422^**NS**^	0.69 ± 0.24^**C**^
*P*- Value**	0.000^**HS**^	0.000^**HS**^		0.000^**HS**^

Descriptive statistics of ridge height reduction (mm) at buccal and palatal/lingual sites for the three groups									
**Descriptives**									
		N	Mean	Std. Deviation	Std. Error	95% Confidence Interval for Mean		Minimum	Maximum
						Lower bound	Upper bound		
Control	Buccal	15	2.07	.32	.07	1.91	2.22	1.50	2.30
	palatal/lingual	15	1.52	.18	.04	1.43	1.61	1.20	1.70
Test1	Buccal	15	1.32	.19	.05	1.22	1.41	1.00	1.60
	palatal/lingual	15	.88	.20	.05	.78	.98	.70	1.20
Test2	Buccal	15	.72	.20	.05	.62	.82	.50	1.10
	palatal/lingual	15	.67	.27	.06	.53	.80	.40	1.10

-* = Overall P-value for Intra-group comparison between the two sides (Paired T- test).

-** =Overall P-value for Inter-group comparison between the three groups (ANOVA test).

- ***Capital letters*** for inter-group comparison between different groups (Tukey Post Hoc test) and the means with different superscripts are statistically significant different at P ≤ 0.05.

- ***HS*** = Highly significant at P ≤ 0.001 S = Statistically significant at P ≤ 0.05.

- ***NS*** = Non-significant P < 0.05.

b) Assessment of the reduction in the alveolar ridge width

Over all levels (1, 3, and 5 mm), the Control group had the greatest reduction (*P-value* ≤ 0.001) in alveolar ridge width (1.78 ± 0.19 mm, 1.12 ± 0.19 mm, 0.94 ± 0.34 mm), with an average of 1.28 ± 0.44 mm across the three levels. Test I have shown (1.46 ± 0.4 mm, 0.86 ± 0.11 mm, 0.64 ± 0.05 mm), resulting in an average of 0.98 ± 0.42 mm (*P-value* ≤ 0.001). Test II recorded the least significant inter-group reduction (*P-value* ≤ 0.001), with an average of 0.76 ± 0.33 mm for the same levels (1.08 ± 0.36 mm, 0.68 ± 0.19 mm, 0.54 ± 0.09 mm). On comparing the three levels for intra-group comparison, there was a statistically highly significant (P-value ≤ 0.001) difference, primarily due to the difference between the 1 mm level (highest mean reduction) and the 5 mm level (lowest mean reduction). A highly significant difference was reported among the three groups regarding alveolar ridge width, with Test II demonstrating the smallest reduction in width (Table 3, Fig. 6b).

**Table 3:** Mean ± SD, intra, and inter group comparison of reductions in alveolar ridge width measurements (mm) at the three levels (1, 3, and 5 mm) for the three studied groups.

	1 mm	3 mm	5 mm	*P*-Value*	Overall
Control	1.78 ± 0.19^**Aa**^	1.12 ± 0.19^**Ab**^	0.94 ± 0.34^**Ab**^	0.000^**HS**^	1.28 ± 0.44^**A**^
Test I	1.46 ± 0.4^**Ba**^	0.86 ± 0.11^**Bb**^	0.64 ± 0.05^**Bb**^	0.000^**HS**^	0.98 ± 0.42^**B**^
Test II	1.08 ± 0.36^**Ca**^	0.68 ± 0.19^**Cb**^	0.54 ± 0.09^**Bb**^	0.000^**HS**^	0.76 ± 0.33^**B**^
P- Value**	0.000^**HS**^	0.000^**HS**^	0.000^**HS**^		0.000^**HS**^

Descriptive statistics of ridge width (mm) for the three groups									
**Descriptives**									
		N	Mean	Std. deviation	Std. Error	95% Confidence Interval for Mean		Minimum	Maximum
						Lower Bound	Upper Bound		
1 mm	Control	15	1.78	0.18	0.05	1.68	1.88	1.50	2.00
	Test I	15	1.46	0.37	0.10	1.25	1.67	1.10	2.10
	Test II	15	1.08	0.34	0.09	0.89	1.27	0.60	1.60
3 mm	Control	15	1.12	0.18	0.05	1.02	1.22	0.80	1.30
	Test I	15	0.86	0.11	0.03	0.80	0.92	0.70	1.00
	Test II	15	0.68	0.18	0.05	0.58	0.78	0.50	1.00
5 mm	Control	15	0.94	0.31	0.08	0.77	1.11	0.60	1.50
	Test I	15	0.64	0.05	0.01	0.61	0.67	0.60	0.70
	Test II	15	0.54	0.08	0.02	0.49	0.59	0.40	0.60

* = Overall P-value for Intra-group comparison between the three levels (ANOVA test).

-** = Overall P-value for Inter-group comparison between the three groups (ANOVA test).

- ***Small letters(a,b,c)*** for intra-group comparison between different levels, while the ***Capital letters(A,B,C)*** for inter-group comparison between different groups (Tukey Post Hoc test) and the means with different superscripts are statistically significant different at P ≤ 0.05.

- ***HS***, Highly significant at P ≤ 0.001 ***S*** = Statistically significant at P ≤ 0.05.

- ***NS***, Non-significant P < 0.05.

#### II.Histological observations

**Fig. 7:**
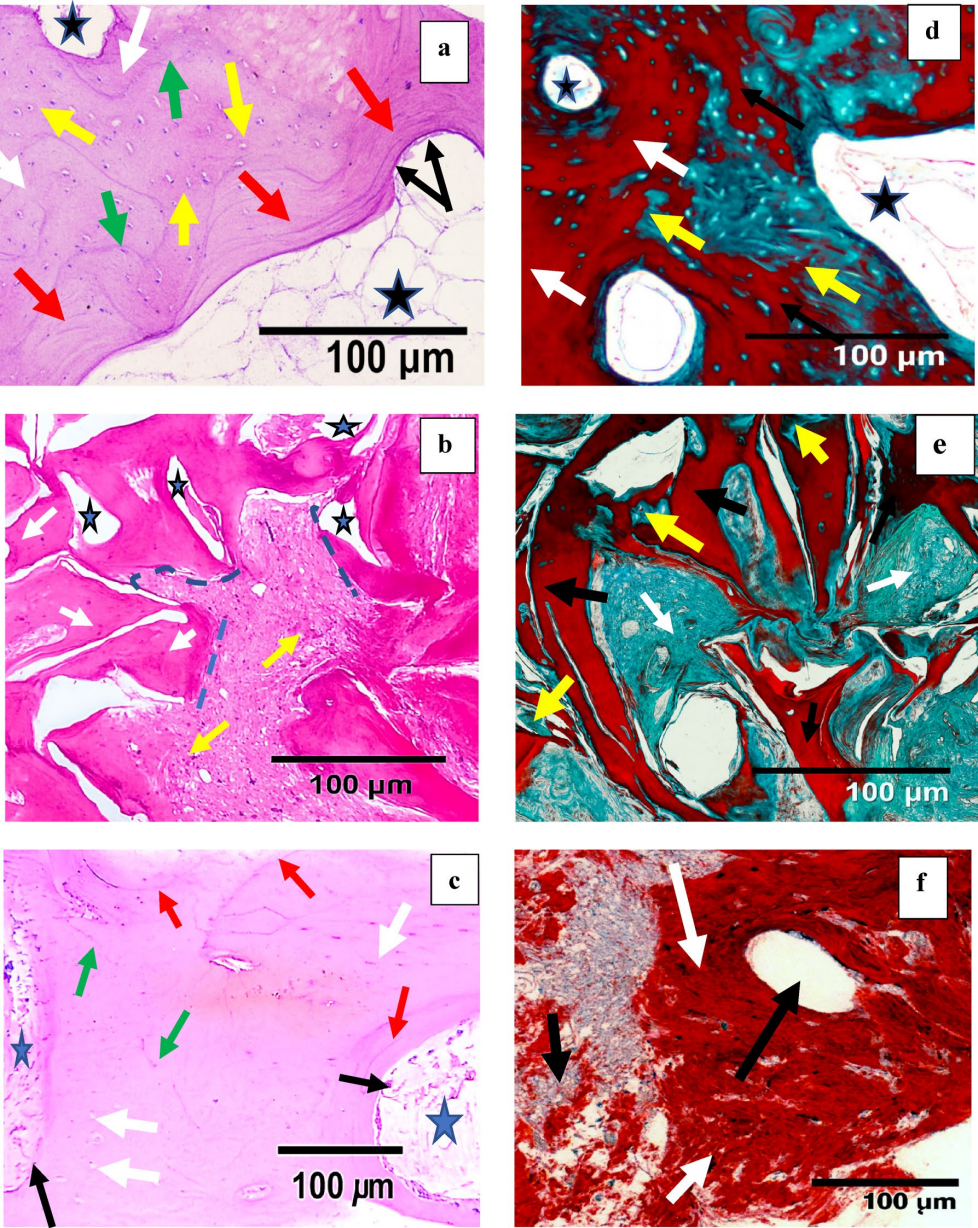
Histological and histomorphometric analysis of bone regeneration across groups with H&E and Masson trichrome staining (**a**) Photomicrograph of Control group showing newly formed bone trabeculae with high number of randomly distributed osteocytes (yellow arrows), other trabeculae showed few or even lacks osteocytes (white arrow), osteoblasts lining the bone surface (black arrows), multiple resting lines (red arrows) accompanied with accentuated reversal lines (green arrow) and fibro-cellular marrow cavities (asterisks). (H&Ex100). (**b**) Photomicrograph of Test I showing newly formed bone trabeculae with areas of empty spaces (asterisks) and few scattered osteocytes (white arrows), other areas exhibited fibrous islands with few inflammatory cells (yellow arrows). No fibrous capsule separation between the graft and the newly formed bone (dotted line), Areas of immature bone appeared in contact with other mature bone trabeculae. (**c**) Photomicrograph of Test II showing osteocytes in the thick well-formed new bone trabeculae (white arrows), osteoblasts lining the bone trabeculae (black arrows), resting lines (red arrows), few reversal lines (green arrows) and bone marrow spaces containing multiple small blood vessels (asterisk), (H&Ex100). (**d**) Photomicrograph of Control group showing areas of mature bone matrix (white arrows), other areas of immature bone matrix (yellow arrows) and collagen fibers surrounding the bone marrow cavities (asterisk), (Masson trichrome × 100). (**e**) Photomicrograph of Test I showing areas of green collagen fibers forming islands (white arrows). Also, small green islands of immature bone (yellow arrows) appeared within the large red mature bone trabeculae (black arrows), (Masson trichrome × 100). (**f**) Photomicrograph of Test II showing large area of connected highly mature bone trabeculae (white arrows) without any collagen fibers or immature matrix in between, marrow cavities (black arrows), (Masson trichrome × 100).

- H & E results:

**Control:** the histological examination revealed formation of new bone trabeculae with different degrees of stainability, multiple resting and reversal lines, and randomly distributed osteocytes. Wide medullary spaces were seen filled with fibro cellular tissue (Fig. 7a).

**Test I:** tissue sections showed new formation of bone trabeculae with areas of empty spaces; other areas exhibited fibrous islands with few inflammatory cells. Areas of immature bone appeared in contact with other mature bone trabeculae (Fig. 7b).

**Test II:** The histological examination showed thick, continuous, well-formed new bone trabeculae with small cellular medullary spaces with multiple blood vessels. A few well-formed and arranged osteocytes in a lamellar pattern accompany multiple resting lines. Osteoblasts appeared to be lining the surface of newly formed bone trabeculae. Few reversal lines were seen (Fig. 7c).

b) Masson trichrome stain results:

**Control:** the formed bone trabeculae exhibited a mixture of mature and immature bone matrix, where mature bone appeared red in color, while immature areas appeared green. The collagen fibers inside the bone marrow appeared green (Fig. 7d).

**Test I:** The sections showed areas of green immature collagen fibers forming islands. Also, small green islands of immature bone appeared within the large red mature bone trabeculae (Fig. 7e).

**Test II:** The formed bone exhibited a large area of connected highly mature bone trabeculae presented as wide red areas without any collagen fibers or immature matrix in between or surrounding marrow cavities (Fig. 7f).

#### III.Histomorphometric results

- Assessment of the area % of new alveolar bone trabeculae

**Fig. 8:**
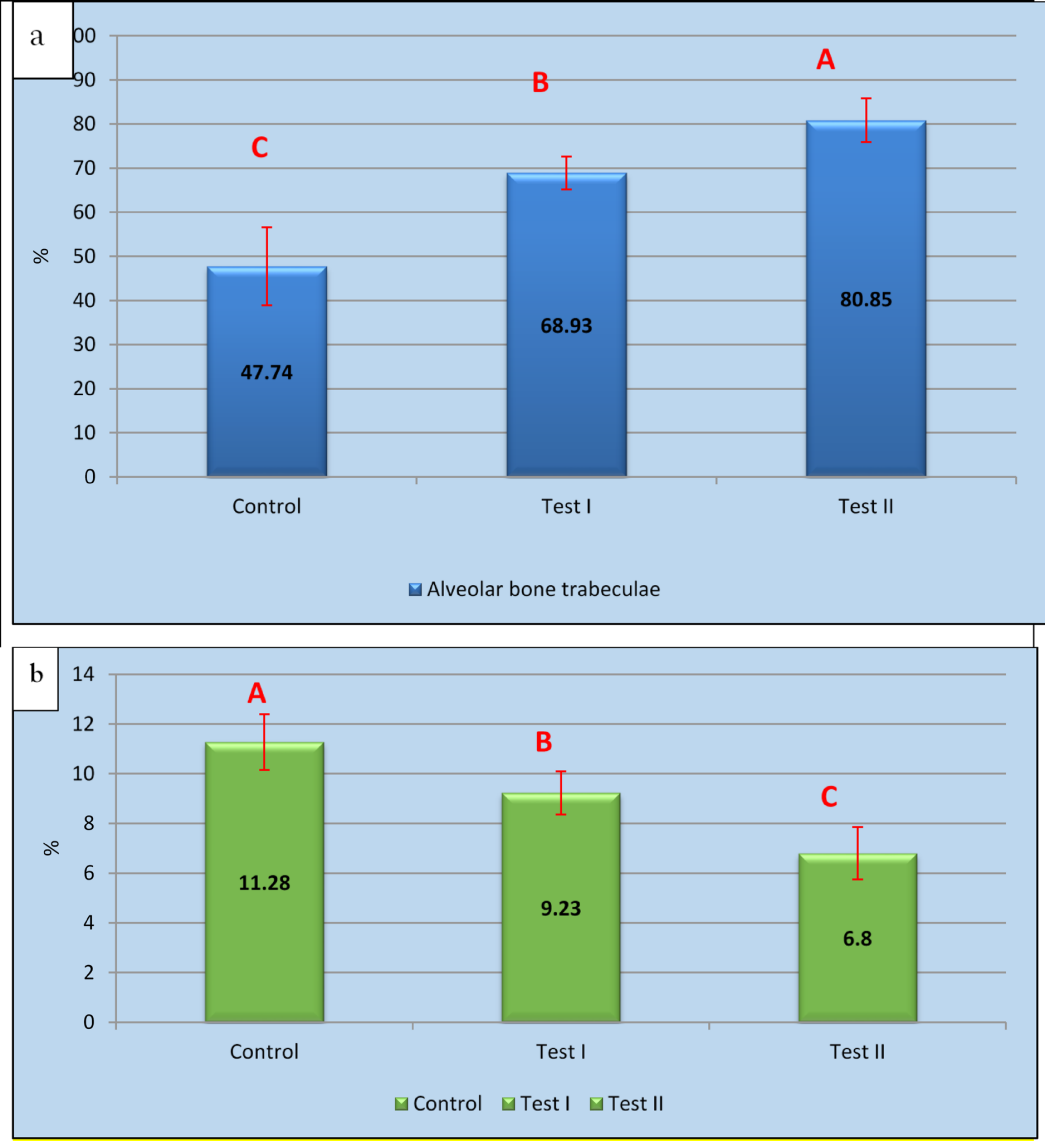
(**a**) Area percentage of newly formed alveolar bone trabeculae. (**b**) Area percentage of immature bone.

Regarding the inter-group comparison of the mean area % of the new alveolar bone trabeculae, it was 47.74 ± 8.88% in Control group, 68.93 ± 3.72% in Test I, and 80.85 ± 4.99% in Test II group showing a significant difference among the study groups (*P-value* ≤ 0.001) with the Control group showing the highest % immature bone while Test II showing the lowest % of immature bone (Table 4, Fig. 8a).

**Table 4:** Mean ± SD and inter group comparison of Histomorphometric results for the three studied groups.

	Area % of new alveolar bone trabeculae	Area % of immature bone
Control	47.74 ± 8.88^**C**^	11.28 ± 1.12^**A**^
Test I	68.93 ± 3.72^**B**^	9.23 ± 0.87^**B**^
Test II	80.85 ± 4.99^**A**^	6.8 ± 1.05^**C**^
*P*- Value**	**0.000**^**HS**^	**0.000**^**HS**^

Descriptive statistics of histomorphometric results for the three studied groups									
Descriptives									
		N	Mean	Std. deviation	Std. error	95% Confidence interval for mean		Minimum	Maximum
						Lower bound	Upper bound		
Alveolar bone trabeculae	Control	15	47.7400	8.87871	2.80770	41.3886	54.0914	33.60	61.40
	Test I	15	68.9300	3.72441	1.17776	66.2657	71.5943	62.40	75.00
	Test II	15	80.8500	4.99049	1.57813	77.2800	84.4200	69.70	86.10
Mature bone	Control	15	6.8000	1.04563	.33066	6.0520	7.5480	5.30	8.50
	Test I	15	9.2300	.87439	.27651	8.6045	9.8555	8.10	10.60
	Test II	15	11.2800	1.12032	.35428	10.4786	12.0814	9.90	13.30

-** = Overall P-value for Inter-group comparison between the three groups (ANOVA test).

***-Superscript letters (A, B, C)*** denote results of inter-group comparisons using Tukey post-hoc testing.

-Means with different letters differ significantly (p ≤ 0.05); means with the same letter are not significantly different.

- HS = Highly significant at P ≤ 0.001 S = Statistically significant at P ≤ 0.05.

- NS = Non-significant P < 0.05.

b. Assessment of the area % of immature bone

The mean immature bone area % was 11.28 ± 1.12% in the Control, 9.23 ± 0.87% in the Test I group, and 6.8 ± 1.05% Test II. The Tukey Post Hoc test revealed a significant difference between the three studied groups. The overall P-value for inter-group comparison among the three groups was statistically highly significant (≤ 0.001), primarily due to the difference between Control (the highest % immature bone) and the Test II group (the lowest % of immature bone). (Table 4, Fig. 8b).

## Discussion

This trial aimed to evaluate PBM-assisted SSS when extraction sockets are grafted with bioactive glass in humans. Alveolar ridge preservation (ARP) is a technique aiming to minimize alveolar ridge dimensions following tooth extraction. It requires incision making and flap lifting that can result in reduction of the blood supply, and causing marginal recession at the adjacent teeth, deficient papillae, swelling, keratinized mucosa loss, postoperative pain increase in patients^28^. Consequently, our protocol began with SSS as an alternative for the management of post-extraction sockets^29^ being a flapless and minimally invasive approach to minimize the inflammation during process of remodeling caused by surgical trauma.

PRF has been extensively used in dentistry for socket preservation, sinus lift, periodontal regeneration, guided tissue regeneration, and wound healing^30^. Femminella et al.^31^ concluded that PRF membrane is effective in reducing the patient’s morbidity, and accelerating wound healing, owing to the growth factors enclosed within the PRF are implicated in promoting cell mitosis and inducing cell differentiation. In addition, the fibrin mesh acts as a scaffold to support the aggregation of growth factors and cytokines, along with promoting their long-term release. Accordingly, this will improve the progress of the healing process. PRF was recently suggested as an appropriate, reliable dressing^32^; thus, it was used in our protocol to seal the sockets and play an important mechanical role in maintaining and serving the graft material.

Autogenous bone graft was one of the pioneer materials used for filling sockets after extraction^33^. Whilst it is considered a benchmark for bone grafting materials, it has many limitations as confined donor sites, unpredictable resorption, and postoperative pain. Therefore, other various bone substitutes are recommended. Bioactive glass was used in various studies to preserve ridge dimensions in post-extraction sockets;^32,33^ bioactive glass particulates appeared to be capable of preserving the ridge contour with further achievement of the osseointegration process of titanium implants^34^.

Bone resorption is not avoided by rapid placement of implants, and significant changes in bone volume have been seen up to the time of four months after immediate implantation^35^. So, crestal bone preservation is considered even before implant placement treatment planning. Sanz et al.,^16^ considered the 4 mm residual bone necessary to prevent excessive resorption and provide sufficient anchorage for implants. This threshold also minimizes the need for extensive augmentation procedures, reducing patient morbidity and treatment time^17^.

Although ridge preservation techniques have been widely used to prevent the loss of bone volume, their effect may vary due to the influence of local and systemic factors. The use of a cheap, noninvasive, fast, safe, and painless tool with no side effects is advised. Therefore, laser PBM has been used as an adjunctive therapy to improve bone repair in our test model. It was immediately applied after tooth extraction and maintained at 48 h interval for 15 days to benefit from the use of laser photobiomodulation during the early stages of tissue repair with regular intervals of multiple irradiations, which was reported to be more efficient than a single dose during tissue regeneration^36^. Laser beam with a wavelength of 980 nm, which was reported to have a longer penetration depth^50^, together with a power density of 0.69 W/cm^2^ for 60 s (energy density of 41.4 J/cm^2^) was applied. to compensate for the attenuation that occurs during tissue penetration, aiming to reach the target tissue with a suitable therapeutic dose as recommended by recent research^23,37^.

Furthermore, A 9-month healing period was selected in this novel clinical trial to evaluate the long-term impact of bioactive glass grafting and PBM on alveolar ridge preservation and bone maturation before implant placement. Previous studies suggested that prolonged healing periods are associated with improved graft integration and a higher ratio of mineralized to non-mineralized tissue^38^. In this respect, our results revealed that the test II group showed the least amount of loss in alveolar ridge dimensions at 9 months.

The lack of significant baseline differences among the study groups demonstrates successful randomization and reinforces the reliability of the findings, suggesting that the observed improvements resulted from the applied interventions rather than inherent group variations.

On comparing the changes between both groups (I and II), significant differences were recorded in favor of PBM therapy. This clinical finding can be attributed to the efficacy of the chosen laser system (980 nm) during the early stages of bone healing and for further calcification^39^. Various in vivo animal studies have indicated that PBM therapy increases the amount and rate of new bone formation^40^. Our findings are also in agreement with Osman et al^41^. who demonstrated that laser-assisted ridge preservation using PRF and/or bone graft resulted in improved ridge width compared to PRF alone, highlighting the beneficial effects of laser therapy on socket healing outcomes.

Different from repairing periodontal defects, the protocol of grafting first and placing the implants later provided a unique opportunity to gain reentry and harvest bone cores. During implantation procedures, trephining out bone cores was done, processed, stained, and examined for evaluation of the tissue response. The histological examination for Test I has shown newly formed bone trabeculae with variable-sized medullary spaces and a high number of randomly arranged osteocytes, while some other trabeculae lacked osteocytes. Also, revealed that no connective tissue capsule separated residual particles from bone tissue. Owing to the biocompatibility of graft particles, evidenced by their embedment in dense connective tissue stroma with minimal inflammatory cell infiltrate, a result that was confirmed by similar studies^42–44^.

In the present study, the PBM group showed increased thickness of bone trabeculae with obvious lamellar structure and proper arrangement of osteocytes within their lacunae, which is indicative of better bone quality and maturity. A higher number of blood vessels was also detected in bone marrow spaces of PBM group samples, in fact, this result is expected due to the particular ability of PBM to modulate the inflammatory process, induce vascular sprouting and, consequently, accelerate the formation of a new bone matrix, favoring the maintenance or even increasing the height and/or thickness of the alveolar bone ridge^45^.

Our histological results were further confirmed using Masson trichrome stain. The qualitative analysis of the tissue samples indicates a more considerable amount of collagen and immature bone matrix in Test I and a more significant amount of mature homogeneously connected trabeculae in the PBM group, without any interrupting areas of collagen fibers or immature matrix. The increased connections between the trabeculae suggest that the repair process is faster and with a better bone quality when compared with the bone tissue of Test I, due to the unique impact of 980 nm laser PBM on cell proliferation, osteogenesis, angiogenesis, and associated gene expression^46,47^.

To provide a quantitative assessment of bone structure in this work, bone histomorphometry was used tool to measure the % of new bone surface area and unmineralized bone area %^48^. The histomorphometric analysis results showed a significantly higher new bone trabecular surface area % in Test II than other groups. In addition to statistically significant lesser areas of un-mineralized bone were observed in this group, denoting the best regenerative process in all groups. These findings can be explained considering previous data suggested that 980 nm laser PBM with suitable power and energy density can speed up the bone repair process, enhance the quality of the newly formed bone, and decrease the time needed for post-extraction implant placement^37^.

Our results can also be related to the use of the unique flat-top handpiece with 40 cm working distance and 0.724 cm^2^ spot area, according to our applied protocol. In this aspect, the flat-top handpiece was reported to have a beam profile with a long working distance that may exceed 100 cm to allow delivery of a homogenous irradiation with nearly unchanged spot area and energy up to the surface of the target tissue. Using this new flat-top handpiece allows irradiating the target tissue with uniform energy density, using somewhat high-power densities, in a shorter time, and with no risk of thermal damage. This would make the application repeatable and not operator sensitive. This technology was recently innovated as an alternative to the Gaussian beam profile that produces an inhomogeneous beam profile, leading to doubling of the power at the beam center, leading to undesired effects or even target tissue damage, and a very weak, mostly ineffective power at the beam peripheries^23^.

To explain our clinical and histological progress, the mechanisms through which PBM works are multifactorial and are involved in several biological actions such as energy metabolism, gene expression, cell proliferation, differentiation, survival, and cell death^49^ As reported by Robling and Turner^50^, two of the earliest events in mechano-transduction signaling, which occur during the first minutes of application of a mechanical stimulus, are an increase in intracellular Ca^2+^ concentrations and ATP. The mechanism involves the modulation of voltage-sensitive calcium channels and calcium stores. Following these signaling events, nitric oxide (NO) acts as a second messenger that plays a role in the response. The NO release results in MAP-kinase signaling and ERK1/2 activation, and finally, the expression of bone matrix genes to promote cell proliferation and cell viability. Differentiation can also be activated by triggering the Wnt and-catenin pathway.

The laser wavelength used in the present study (980 nm), with suitable parameters, was reported to produce its photobiomodulation effect by stimulating the light-sensitive ion channels in the cell membrane with the subsequent release of ROS, nitric oxide, calcium ions, and cyclic AMP (cAMP), which then results in the activation of transcription factors. Laser PBM can also modulate inflammation in bone defects and stimulate the deposition of granulation tissue and newly formed bone tissue, as a consequence of angiogenic gene expression^37,51^. In addition, it was reported that PBM using 980 nm diode laser enhances the pre-osteoblast differentiation through activation of Wnt signaling and activation of Smads 2/3-βcatenin pathway^52^. Moreover, PBM can modulate cyclooxygenase-2 (COX2) and vascular endothelial growth factor (VEGF) expression during the initial phase of bone regeneration^53–55^.

The present study gives a novel clinical investigation by integrating PBM with bioactive glass in socket seal surgery for alveolar ridge preservation in clinical settings. To the best of our knowledge, this is the first randomized clinical trial to assess the adjunctive use of PBM with bioactive glass in socket seal surgery for implant site development. This dual-modality approach not only enhances bone quality, evidenced by superior histological features as well-organized, thicker bone trabeculae and increased vascularity, but also minimizes alveolar ridge resorption compared to the conventional protocol of SSS.

Our findings ultimately introduce a unique, minimally invasive protocol that influences the regenerative synergy between PBM and bioactive glass, offering a clinically practical approach to enhance both the quality and quantity of alveolar bone following tooth extraction.

## Conclusion

The combined use of PBM and bioactive glass following tooth extraction hastened bone formation and preserved alveolar crest height, suggesting a promising role in socket preservation. Our protocol can be successfully used for the SSS strategy, being a non-invasive and effective tool in bone repair, as evidenced by our radiographic and histologic findings.

### Limitations of the study

These findings should be interpreted cautiously, considering the trial’s limitations. A notable shortcoming is the absence of patient-reported experience, such as pain scores and post-operative satisfaction. Additionally, the smaller sample size, the lack of early and intermediate evaluation time points (4–12 weeks and 3–6 months) limited the ability to capture the dynamic effects of PBM on bone remodeling and healing. Future studies should incorporate these intervals for a more comprehensive understanding of PBM’s temporal impact.

This study also has limitations that should be acknowledged. One important shortcoming is the absence of a PBM-only group, which would have allowed clearer assessment of the independent effect of laser irradiation on socket healing. Future research should include sham PBM with matched visits to reduce performance bias and achieve complete blinding of both participants and clinicians. Longer-term follow-up is also required to assess the impact on ridge morphology, peri-implant soft tissue health, and implant success rates.

Furthermore, exploring different biomaterial combinations or modifying PBM parameters may help to optimize treatment protocols for alveolar ridge preservation.

### Recommendations

Further studies are necessary to investigate the cellular and molecular mechanisms involved in PBM therapy, as well as the impact on bone osseointegration, also the primary and long-term stability of dental implants. Integrating longitudinal densitometric analysis using Hounsfield units (HU) based assessments in future studies could enhance understanding of bone remodeling dynamics.

## Data Availability

The data that support the findings of this study are available on request from the corresponding author, email: [noramohammed.26@azhar.edu.eg](mailto:noramohammed.26@azhar.edu.eg).
